# A Cross-Layer Routing Protocol Based on Quasi-Cooperative Multi-Agent Learning for Multi-Hop Cognitive Radio Networks

**DOI:** 10.3390/s19010151

**Published:** 2019-01-03

**Authors:** Yihang Du, Chun Chen, Pengfei Ma, Lei Xue

**Affiliations:** 1 National University of Defense Technology, Shushan District, Hefei 230000, China; yuri_wolfdyh@163.com (Y.D.); xwdxh1965@163.com (L.X.); 2 Army Academy of Artillery and Air Defense, Shushan District, Hefei 230000, China; xiaoma.2002@163.com

**Keywords:** cognitive radio, cross-layer routing protocol, experience replay, quasi-cooperative multi-agent learning, stochastic game

## Abstract

Transmission latency minimization and energy efficiency improvement are two main challenges in multi-hop Cognitive Radio Networks (CRN), where the knowledge of topology and spectrum statistics are hard to obtain. For this reason, a cross-layer routing protocol based on quasi-cooperative multi-agent learning is proposed in this study. Firstly, to jointly consider the end-to-end delay and power efficiency, a comprehensive utility function is designed to form a reasonable tradeoff between the two measures. Then the joint design problem is modeled as a Stochastic Game (SG), and a quasi-cooperative multi-agent learning scheme is presented to solve the SG, which only needs information exchange with previous nodes. To further enhance performance, experience replay is applied to the update of conjecture belief to break the correlations and reduce the variance of updates. Simulation results demonstrate that the proposed scheme is superior to traditional algorithms leading to a shorter delay, lower packet loss ratio and higher energy efficiency, which is close to the performance of an optimum scheme.

## 1. Introduction

Cognitive radio is a technology that is used to settle the problem of spectrum scarcity by enabling secondary users (SUs) to access the licensed spectrum of primary users (PUs) in a dynamic and non-interfering manner. It mediates the contradiction between the regulation and frequency utility via time and spatial multiplexing [1]. One of the major challenges in the design of cognitive radio networks (CRNs) is radio resource management, which efficiently handles the spectrum mobility and quality of service (QoS) requirements of different services and different nodes [2]. Consequently, techniques for resource management have been receiving considerable attention [3,4].

However, most research in the CRN field has focused on the direct transmission network. The research community has focused on applying the cognitive paradigm in multi-hop networks to supply more spectrum resources for a range of applications [5,6]. To fully study the characteristics of multi-hop CRN, it is critical to coordinate route selection with radio resource management and design a cross-layer routing protocol for high-spectrum utility. The regional difference in a multi-hop path leads to the difference in the frequency band for all SUs [7]. The design of efficient and robust spectrum-aware routing protocol is challenging due to the absence of topology information and spectrum dynamics in CRNs.

Traditional protocols used decompose the cross-layer design problem into two sub-problems including route selection and resource management. The sub-problems were optimized separately to reduce the system calculation complexity. Ding et al. [8] proposed a distributed and localized scheme for joint relay selection and channel assignment. A cooperative strategy for the optimization problem based on real-time decentralized policies was studied. However, full cooperation was difficult to achieve for SUs in CRN due to its uncertainty. A centralized routing protocol was proposed by Lai et al. [9], which expanded the paths hop-by-hop and discarded unnecessary paths. Then the resource management problem was formulated into an optimization problem with the aforementioned objective and restriction. Nevertheless, the centralized method was more complex in calculation and less flexible than distributed schemes. An economic framework was presented by Amini et al. [10] that integrated route selection and spectrum assignment for the improvement of QoS performance in cognitive mesh networks. The decomposed model allowed for a decentralized implementation of routing and spectrum allocation, which increased the robustness of the algorithm. These works aimed at finding a fixed path from the source nodes to the destination nodes [11]. However, fixed routing strategies have two disadvantages: priori knowledge of topology and spectrum dynamics is required, which is hard to obtain in multi-hop CRN, and fixed protocols may become invalid due to the dynamic and uncertain nature of frequency.

In order to realize real cognition, a cognitive radio (CR) should be capable of learning and reasoning [12]. Machine learning technology has been widely used to address dynamic channel statistics in CRNs. Raj et al. [13] proposed a two-stage reinforcement approach to select a channel via a multi-armed bandit, and then predict how long the channel would remain unoccupied. Its sensing was more energy efficient and achieved higher throughput by saving on spectrum detection. Al-Rawi et al. [14] developed the cognitive radio Q-routing algorithm, which adopted reinforcement learning (RL) method to enable flexible and efficient routing decisions. Single-agent reinforcement learning with limited capacity was adopted by Raj et al. [13] and Al-Rawi et al. [14]. Nevertheless, learning strategy with multiple agents is more suitable for solving complicated problem in multi-hop CRNs. Multi-agent learning approaches in CRN have drawn the interest of researchers for their superior performance. A conjecture-based multi-agent Q-learning scheme was presented by Chen et al. [15] to execute power adaption in a partially observable environment. However, route selection in multi-hop CRN was not considered in this work. Pourpeighambar et al. [16] modeled the routing problem as a stochastic game (SG). Then, the SG was solved through a non-cooperative multi-agent learning method in which each secondary user (SU) speculated other nodes’ strategies without acquisition of global information. The current conjecture belief was determined only by the last one in [16], which caused strong correlations between the samples. Power adaption was not considered when solving the routing problem, which would influence power efficiency and the routing decision.

In our previous work [17], we designed a single-agent based intelligent joint routing and resource assignment scheme for CRN to achieve the maximum cumulative rewards. In this paper, we adopt a quasi-cooperative multi-agent learning scheme for routing and radio resource management, which is more efficient than the single-agent strategy in multi-hop CRN. The scheme tries to achieve the lowest end-to-end delay and improve energy efficiency with finite information exchange between competing SUs. Our contributions are summarized as follows:

(i) In order to jointly capture the end-to-end latency and power efficiency, a comprehensive utility function is designed to form a reasonable tradeoff between the two as well as accommodate the maximal transmission latency requirement. Queuing theory is adopted to analyze single-hop latency and provide a theoretical basis for our cross-layer routing protocol design.
(ii) A quasi-cooperative multi-agent learning framework is presented to solve the cross-layer design problem where every SU node speculates other nodes’ strategies from finite information exchange with the previous nodes. The convergence of the quasi-cooperative learning scheme is proven.
(iii) For the purpose of further enhancing performance, experience replay is applied to the update of conjecture belief, which allows for greater data efficiency by using the historical conjectures and breaks the correlations to reduce the variance of updates.

The remainder of this paper is organized as follows: the system model is presented in Section 2. Section 3 models the cross-layer design problem as a SG. The quasi-cooperative multi-agent learning scheme for the cross-layer routing protocol is proposed in Section 4. Section 5 demonstrates the simulation results. Finally, the paper is concluded in Section 6. In addition, summary of acronyms used in this paper is listed in Table 1.

## 2. System Model

**Figure 1:**
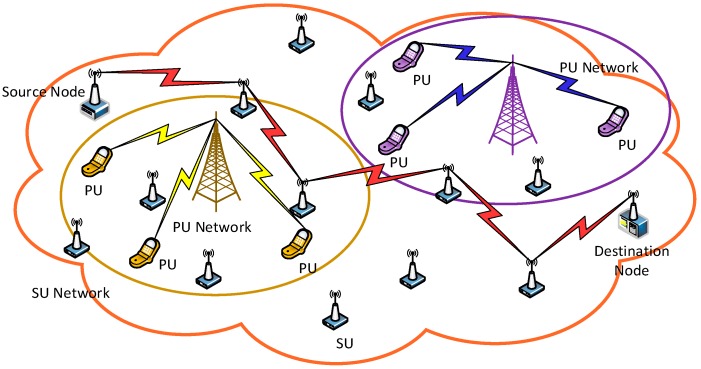
Multi-hop cognitive networking scenarios.

A multi-hop CRN comprising M PUs and N SUs is considered. Specific spectrum bands are assigned to PUs according to the fixed spectrum allocation regulation. SUs occupy no licensed channels and transmit data opportunistically when finding that frequency bands are not held by the PUs. Every SU node $n_{i}$ has a set of available channels that consist of Data Transmission Channel (DTC) and Common Control Channel (CCC). The DTC is used for data transmission and SU $n_{i}$’s DTC is represented as $C_{i}=\left\{c_{1},c_{2},\ldots ,c_{m}\right\}$; whereas the CCC is used by SUs to exchange the negotiation. At any time, a directed communication link can be constructed between SU $n_{i}$ and $n_{j}$ if at least one common DTC $c\in C_{i}\cap C_{j}$ exists. The network model is shown in Figure 1. In the networking scenarios, the multi-hop CRN coexists with two centralized Primary User (PU) networks. As Figure 1 shows, the source SU generates data packets and sends them to the destination node in multi-hop manner through intermediate SUs. Each PU communicates with the PU base station using a licensed frequency, and intermediate SUs in each hop transmit data via the PU channel when the spectrum band is idle.

We assume that every node in the multi-hop CRN maintains a queuing buffer for the storage of SU packets. For data flow f generated from source node $n_{s}$, packet arrivals are considered as a stationary Bernoulli process with mean $\lambda_{s}^{f}$ that is independent and identical at all time slots [18]. In addition, every SU node has respective queues for each traffic flow, and the packet arrival process of every data stream is independent from each other.

The PUs’ occupation model is considered as an ON/OFF process [19]. The probability density function (PDF) of the OFF periods (when PUs do not occupy the channel) is shown as follows:

(1) $$f\left(t\right)=\left\{ \begin{array}{cc} \theta_{d}e^{-\theta_{d}t} & t\geq 0\\ 0 & t<0 \end{array}\right.,$$

where $\theta_{d}$ is departure rate of the PU, and f(t) represents the idle probability of PU channel at time step *t*. Accordingly, the probability that idle period of PU channel is longer than duration τ is denoted as:

(2) $$P\left(t\geq \tau \right)=\int_{\tau }^{\infty }f\left(t\right)dt=e^{-\theta_{d}\tau },$$

where τ is the duration that PU channel is idle. Then, the probability of the collision between PU and SU in the duration τ (i.e., the probability of PU reoccupying the spectrum band in the duration τ) is given by:

(3) $$P_{collision}=1-P\left(t\geq \tau \right)=1-e^{-\theta_{d}\tau }.$$

We use the analysis described in [20] to calculate the PU departure rate $\theta_{d}(\mu ,\sigma )$ with expected mean μ and deviation σ. The spectrum statistic that is parameterized in [21] changes slowly so that it is assumed to be almost static in this work. Every SU can only locate its own position through some positioning equipment.

## 3. Formulation for Joint Design Problem

In this section, to minimize transmission latency and ensure power efficiency, a comprehensive utility function is designed to create a reasonable tradeoff between the two with a delay constraint. Then a measurement called responsibility rating is introduced for power assignment and reducing the action space of agents. On the basis of the above considerations, the cross-layer routing problem is modeled as a SG.

### 3.1. Comprehensive Utility Function

To guarantee the QoS performance of cross-layer routing, a comprehensive utility function is applied to integrate transmission latency and energy efficiency. A multi-hop network must reduce the transmission latency so that the packet loss rate decreases and the routing stability improves. In addition, the requirement of high power efficiency is also a critical factor for energy-sensitive applications. For instance, grid monitoring and control applications only have limited data to send but demand real-time delivery [22]. Energy-constrained networks that traditionally operate powered by batteries are sensitive to power consumption and thus face an inherent challenge in energy efficiency, but are not sensitive to latency. However, some high-level services such as video and audio demand both real-time data transmission and high power efficiency [23]. Low transmission latency demands high power consumption, whereas excessive energy conservation may cause poor QoS performance and result in a long end-to-end delay. There is an inherent tradeoff between the transmission latency and energy consumption. Therefore, a utility function is designed that jointly captures the end-to-end delay and energy efficiency while making a reasonable tradeoff between the two. The resultant design is as follows:

(4) $$r_{i}^{t}={-\log}_{2}\left(\alpha \cdot u_{TD,i}+\beta \cdot u_{PCR,i}\right),$$

where $u_{TD,i}$ accounts for the single-hop transmission latency (TL), and $u_{PCR,i}$ denotes power consumption ratio (PCR) for $SU_{i}$, which will be elaborated in following sections; and α and β are parameters that adjust the tradeoff between transmission delay and energy efficiency, respectively. A larger α increases the weight of the transmission delay in the utility function, whereas larger β emphasizes the power consumption, and vice versa. The logarithmic operation is used for compressing large values of $\alpha \cdot u_{TD,i}+\beta \cdot u_{PCR,i}$ to a relatively small range. We can see from Equation (4) that the larger the TL or PCR is, the lower the utility function $r_{i}^{t}$ becomes. This results in little reward for the agent so that it will explore more efficient actions to achieve minimal latency and energy expenditure.

#### 3.1.1. Transmission Delay

The transmission delay consists of queuing waiting time and data transmission time. Firstly, the queuing waiting time is computed based on the packet arrival and service rates of every SU in a multi-hop CRN [18]. The calculation method of queuing waiting time $\delta_{i}$ was proposed in [24], where the packet arrival and service rates of every SU were used to compute the queuing waiting time. In our work, we further combine this method with the concept of strategy $\zeta_{k}(s_{k},\;a_{k})$ in RL to obtain the latency for queuing in SU’s buffer. A packet from flow f is placed in the SU $n_{i}$’s queuing buffer at time step *t* if:

(1) One of the node $n_{i}$’s neighboring nodes selects node $n_{i}$ as its next hop and transmits data via an available channel c;
(2) Channel c is idle during time step *t*, and
(3) There is at least one packet in the queue of the preceding SU node to communicate with node $n_{i}$

Therefore, the arrival rate at SU $n_{i}$ is the joint probability of all events above, which can be represented as the product of these events’ probabilities due to the independence among themselves:

(5) $$\lambda_{i}^{t}=\sum_{f\in F}\sum_{k\in L_{i}}\sum_{c\in C_{k}}\zeta_{k}(s_{k},\;a_{k})\cdot \frac{\lambda_{f}}{\mu_{f}}\cdot \alpha_{c}\cdot \left(1-P_{ki}^{out}\right),$$

where F is the set of all data streams; $L_{i}$ is the set of $SU_{i}$’s previous nodes that select node $n_{i}$ as their next hop; $C_{k}$ is the set of available channels of node $n_{k}\in L_{i}$; $\zeta_{k}(s_{k},\;a_{k})$ is the strategy of node $n_{k}\in L_{i}$, i.e., the probability of node $n_{k}$ choosing action $a_{k}$, which corresponds to the next intermediate node $n_{i}$ and the operating channel $c\in C_{k}$; $\lambda_{f}$ is the arrival rate of data flow f; and $\mu_{f}$ is its service rate. The probability that the queue has at least one packet can be represented as $\lambda_{f}/\mu_{f}$ according to the theory of discrete time Markov chain. $\alpha_{c}$ is the probability that channel c is idle and $P_{ki}^{out}$ represents the outage probability of the link between node $n_{k}$ and $n_{i}$.

Like the arrival rate, the service rate is equal to the probability of transmitting a packet successfully at SU $n_{i}$, which occurs if:

(1) $SU_{i}$ selects node $n_{j}$ as its next hop and transmits data via an available channel $c^{\prime }$, and
(2) Channel $c^{\prime }$ is idle during time step *t*.

Accordingly, the service rate at SU $n_{i}$ can be calculated as the product of these two events’ probability:

(6) $$\mu_{i}^{t}=\sum_{f\in F}\sum_{j\in N_{i}}\sum_{c^{\prime }\in C_{i}}\zeta_{i}(s_{i},\;a_{i})\cdot \alpha_{c}\cdot \left(1-P_{ij}^{out}\right),$$

where $N_{i}$ is the set of $SU_{i}$’s neighboring nodes; $C_{i}$ is the set of node $n_{i}$’s available channels; $\zeta_{i}(s_{i},\;a_{i})$ is the strategy of node $n_{i}$, i.e., the probability of node $n_{i}$ choosing action $a_{i}$, which corresponds to the next intermediate node $n_{j}$ and the data transmission channel $c^{\prime }\in C_{i}$; and $P_{ij}^{out}$ represents the outage probability of link between node $n_{i}$ and $n_{j}$.

As discussed above, the arrival and service processes of every SU are Bernoulli processes with rates $\lambda_{i}^{t}$ and $\mu_{i}^{t}$, respectively. This queuing system is modeled as a Geo/Geo/1 queue [25]. Consequently, the queuing waiting time of SU $n_{i}$ is calculated as:

(7) $$\delta_{i}=\frac{\lambda_{i}^{t}}{\mu_{i}^{t}\left(\mu_{i}^{t}-\lambda_{i}^{t}\right)}.$$

Large packet size, interference of PUs and bandwidth constraint will lead to limited channel capability. So, the data transmission time has to be considered. The data transmission time of SU $n_{i}$ is defined as:

(8) $$\tau_{i}=R_{packet}/\left[B\cdot \log_{2}\left(1+\frac{h_{ijc}p_{i}}{\vartheta +\phi_{ijc}^{PU}}\right)\right],$$

where $R_{packet}$ is the packet size, B is the bandwidth of DTC, $h_{ijc}$ represents the channel gain between the node $n_{i}$ and $n_{j}$, $\phi_{ijc}^{PU}$ denotes the PU-to-SU interference at the receiver node $n_{i}$, and ϑ is the additive white Gaussian noise (AWGN) power. Consequently, the transmission delay is calculated as:

(9) $$u_{TD,i}=\delta_{i}+\tau_{i}=\frac{\lambda_{i}^{t}}{\mu_{i}^{t}\left(\mu_{i}^{t}-\lambda_{i}^{t}\right)}+R_{packet}/\left[B\cdot \log_{2}\left(1+\frac{h_{ijc}p_{i}}{\vartheta +\phi_{ijc}^{PU}}\right)\right].$$

#### 3.1.2. Power Consumption Ratio

The power consumption ratio (PCR) is the energy consumption when obtaining unit throughput. It is proposed to describe power efficiency. A low PCR means that the cognitive node expends less energy when transmitting the same size of SU packet data, which represents high energy efficiency. PCR is given by:

(10) $$\varepsilon_{i}=p_{i}/B\cdot \log_{2}\left(1+\frac{h_{ijc}p_{\psi_{i}}}{\vartheta +\phi_{ijc}^{PU}}\right),$$

where $p_{i}$ is the transmission power for node $n_{i}$.

### 3.2. Responsibility Rating

For power efficiency and PU protection, power assignment is considered in this work. However, the action space will be fairly large if we treat power assignment as actions in the joint optimization problem. Huge action space results in intensive computation complexity and low learning efficiency due to the maximum calculation in Q-value updating [17]. In this case, the concept called responsibility rating was introduced in our previous work [17].

SU should improve the transmitting power in its next transmission for reducing the average TL if much time has been wasted in the current transmission. If the latency of the current data transmission is sufficiently short, then the power should be lessened at the next time step to decrease energy expenditure. Based on this principle, the responsibility rating of SU $n_{i}$ at time step *t* is given by:

(11) $$\psi_{i}^{t+1}=\left\{ \begin{array}{ll} \psi_{i}^{t}+1 & u_{TD,i}>\Lambda_{i,t}^{*}\\ \psi_{i}^{t}-1 & u_{TD,i}\leq \Lambda_{i,t}^{*} \end{array}\right.,$$

where responsibility rating $\psi_{i}^{t}$ is a nonnegative integer corresponding to one of the transmission power levels. In addition, if $\psi_{i}^{t}=\max\left\{\psi_{i}^{t}\right\}$ and $u_{TD,i}>\Lambda_{i,t}^{*}$, then $\psi_{i}^{t+1}=\psi_{i}^{t}$; if $\psi_{i}^{t}=0$ and $u_{TD,i}\leq \Lambda_{i,t}^{*}$, then $\psi_{i}^{t+1}=0$. $u_{TD,i}$ denotes the single-hop TL of SU $n_{i}$, and $\Lambda_{i,t}^{*}$ is the average value of $u_{TD,i}$ at time step *t*, which can be calculated in a progressive form via historical information:

(12) $$\Lambda_{i,t}^{*}=\Lambda_{i,t-1}^{*}+\frac{1}{t}\left(u_{TD,i}-\Lambda_{i,t-1}^{*}\right).$$

Every responsibility rating $\psi_{i}^{t}$ matches a transmission power $p_{i}\;(p_{\min}\leq p_{i}\leq p_{\max})$, and the association is given by:

(13) $$p_{i}\left(\psi_{i}^{t}\right)=\left(1-\frac{\psi_{i}^{t}}{\left|\Psi_{i}\right|}\right)p_{\min}+\frac{\psi_{i}^{t}}{\left|\Psi_{i}\right|}p_{\max},$$

where $\left|\Psi_{i}\right|$ represents the size of $\left\{\psi_{i}^{t}\right\}$, and $p_{\min}$ and $p_{\max}$ are the minimal and maximal value of the transmit power, respectively. The responsibility rating not only adjustments transmitting power for high energy efficiency but compresses huge action space to reduce the computation load.

### 3.3. Problem Definition

In this part, the cross-layer design problem is formulated as stochastic learning processes featured by quasi-cooperative games. The quasi-cooperative game is defined by a tuple ${\langle S_{i},A_{i},T_{i},R_{i}\rangle }_{i=1}^{N}$, where $S_{i}$ is SU $n_{i}$’s state space, $A_{i}$ represents SU $n_{i}$’s actions space, $T_{i}$ is the state transferring probability set, and $R_{i}:S_{i}\times A_{i}\mapsto ℜ$ specifies the reward received by SU $n_{i}$ at $s_{i}\in S_{i}$ when taking action $a_{i}\in A_{i}$. SU’s states, available actions and instantaneous reward are precisely defined as follows:

#### 3.3.1. States

For SU $n_{i}$, the node state at time step *t* is defined as:

(14) $$s_{i}^{t}=\left\{\rho_{i},\;\psi_{i}^{t}\right\},$$

where $\psi_{i}^{t}$ is the responsibility rating of SU $n_{i}$, and $\rho_{i}\in \left\{0,1\right\}$ is the Signal-to-Interference plus Noise Ratio (SINR) indicator that indicates whether the SINR $\gamma_{i}$ of SU $n_{i}$ is above or below the threshold $\gamma_{th}$:

(15) $$\rho_{i}=\left\{ \begin{array}{ll} 1, & \mathrm{if}\;\gamma_{i}\geq \gamma_{th}\\ 0, & \mathrm{otherwise} \end{array}\right.,$$

where $\gamma_{i}=h_{ijc}p_{\psi_{i}}/\left(\vartheta +\phi_{ijc}^{PU}\right)$, $p_{\psi_{i}}$ is the transmitting power of SU $n_{i}$, $h_{ijc}$ represents the channel gain between node $n_{i}$ and $n_{j}$, $\phi_{ijc}^{PU}$ denotes the PU-to-SU interference at $n_{i}$, and ϑ is the AWGN power. In addition, a learning episode of SU $n_{i}$ terminates when $\rho_{i}=0$, i.e., $s_{i}^{t}=\left\{0,\;\psi_{i}^{t}\right\}$ is the terminal state in the Markov chain.

#### 3.3.2. Actions

For the joint route selection and resource management problem, an action at time step *t* is defined as $a_{i}^{t}=\left\{n_{j},\;c_{i},\;p_{\psi_{i}}\right\}$, where $n_{j}$ is the next relay node in SU $n_{i}$’s neighboring nodes set, $c_{i}\in C_{i}$ denotes the DTC of node $n_{i}$, and $p_{\psi_{i}}$ is the transmission power described in Equation (13). Assume that the size of neighboring node set is J, the DTC of node $n_{i}$ consists of C channels, and the transmitting power is divided into P levels. The size of state space is 2 ($\rho_{i}=0\;\mathrm{or}\;1$) and action space is J×C×P if the power assignment is set as the action. By applying the responsibility rating to the cross-layer design, the size of state rises to 2×P, while the action space size becomes J×C×1. Therefore, a tradeoff occurs between the size of state space and action space, which reduces the calculation complexity when updating the Q-values by compressing huge action space while controlling the size of state space in case of dimension curse.

#### 3.3.3. Rewards

$R_{i}^{t}(s_{i},a_{i},a_{-i})$ is the instantaneous reward when SU $n_{i}$ performs action $a_{i}$ in $s_{i}$ and other competing SUs execute actions $a_{-i}$. The considered reward function at time step *t* is calculated as follows:

(16) $$R_{i}^{t}(s_{i},a_{i},a_{-i})=\left\{ \begin{array}{ll} r_{i}^{t}(s_{i},a_{i},a_{-i}), & \mathrm{if}\;u_{TD,i}\leq \kappa_{th}\\ 0, & \mathrm{otherwise} \end{array}\right.,$$

where $a_{-i}=(a_{1},\ldots ,a_{i-1},a_{i+1},\ldots ,a_{N})\in A_{-i}=\prod_{j\in N\backslash \left\{i\right\}}A_{j}$ is other SUs’ action vector, $r_{i}^{t}(s_{i},a_{i},a_{-i})$ is the utility function defined in Equation (4), and $\kappa_{th}$ represents the maximal transmission delay threshold between SU nodes. If TL is smaller than the maximal delay threshold, the transmission is effective and the instantaneous reward is equal to the utility function. Otherwise, the agent will receive no reward.

As described in Equations (5) and (6), SU $n_{i}$ needs its own strategy $\zeta_{i}(s_{i},\;a_{i})$ and the strategies of the previous nodes $\zeta_{k}(s_{k},\;a_{k})$ to calculate the transmission delay and the average reward. Since every SU node only needs local observations and information exchange with the previous nodes instead of mutual information sharing between competing SU nodes, the problem is formulated as quasi-cooperative stochastic games, which is formally defined as:

(17) $$\begin{array}{ll} & \underset{a_{i}\in A_{i}}{\max}R_{i}^{t}(s_{i},a_{i},a_{-i})\\ s.t. & u_{TD,i}\leq \kappa_{th} \end{array}$$

Every agent chooses the action of route selection and spectrum access in terms of the strategy $\zeta_{i}(s_{i},\;a_{i})$, which matches the definition of mixed-strategy game. Moreover, each SU cannot acquire the global information of competing SUs due to the uncertainty of multi-hop CRN. One of the significant characteristics in Mixed-Strategy Nash Equilibrium (MSNE) is that the players cannot obtain their opponents’ strategies in advance. In other words, MSNE is a rational countermeasure when the strategies of other players are uncertain. Therefore, MSNE is adopted to solve the quasi-cooperative stochastic game [26].

**Definition.**  *A set of M strategies $\left(a_{i}^{*},a_{-i}^{*}\right)$ is an MSNE if, for every SU $n_{i}\in M$:*

(18) $$R_{i}(a_{i}^{*},a_{-i}^{*})\geq R_{i}(a_{i},a_{-i}^{*}),\;for\;all\;a_{i}\in A_{i}.$$

In the following part, we study the method of speculating competing SUs’ strategies only using information exchange with the previous nodes for SU $n_{i}$, and solve quasi-cooperative stochastic game through multi-agent Q-learning.

## 4. Joint Routing and Resource Management with Conjecture Based Multi-Agent Q-Learning

In order to introduce the quasi-cooperative multi-agent learning scheme, a brief introduction to multi-agent Q-learning is provided in Section 4.1. In Section 4.2, the Equal Reward Time-slots based Conjectural Multi-Agent Q-Learning (ERT-CMQL) is presented to solve the cross-layer routing problem. Then, the analysis and proof of its convergence is outlined in Section 4.3.

### 4.1. Multi-Agent Q-Learning

Among various algorithms in the RL framework, Q-learning is a practical approach adopting Q-value. Q-value is the total expected discounted reward for the pair of state-action and describes the value of choosing a particular action in a given state. It weights and ranks the probabilities of different actions, i.e., the action with a higher Q-value is more valuable and given higher selection probability, and vice versa. To achieve this object, the Boltzmann distribution is used to calculate the probability of choosing action $a_{i}$ at time slot *t*:

(19) $$\zeta_{i}^{t}\left(s_{i},a_{i}\right)=\frac{e^{Q_{i}^{t}\left(s_{i},a_{i}\right)/\eta }}{\sum_{b\in A_{i}}e^{Q_{i}^{t}\left(s_{i},b\right)/\eta }},$$

where $Q_{i}^{t}(s_{i},a_{i})$ is the Q-value for the pair of state-action $\left(s_{i},a_{i}\right)$ at time step *t*, η is a positive number called the temperature. The larger the temperature is, the more balanced the probability of action selection becomes, and vice versa.

*M* players are considered and every player is fitted with a Q-learning agent that learns its own strategy through limited cooperation with other agents. Thus, the Q-value is updated according to multi-agent Q-learning rule:

(20) $$\begin{array}{cc} Q_{i}^{t+1}(s_{i},a_{i}) & =(1-\alpha )Q_{i}^{t}(s_{i},a_{i})+\alpha \left[E\left[R_{i}(s_{i},\zeta_{i},\zeta_{-i})\right]+\beta \underset{b_{i}\in A_{i}}{\max}Q_{i}^{t}({s^{\prime }}_{i},b_{i})\right]\\ & =Q_{i}^{t}(s_{i},a_{i})+\alpha \left[E\left[R_{i}(s_{i},\zeta_{i},\zeta_{-i})\right]+\beta \underset{b_{i}\in A_{i}}{\max}Q_{i}^{t}({s^{\prime }}_{i},b_{i})-Q_{i}^{t}(s_{i},a_{i})\right] \end{array}$$

where α∈[0, 1) is the learning rate, and β is the discount factor, and $E\left[R_{i}(s_{i},\zeta_{i},\zeta_{-i})\right]$ is the expected reward for SU $n_{i}$ at time slot *t* considering other M−1 competing SUs. The variation of Q-value is proportional to the expected reward plus the difference between the target and evaluated Q-value. The detailed definition of $E\left[R_{i}(s_{i},\zeta_{i},\zeta_{-i})\right]$ is given by:

(21) $$E\left[R_{i}(s_{i},\zeta_{i},\zeta_{-i})\right]=\sum_{(a_{i},a_{-i})\in A}\left[R_{i}(s_{i},a_{i},a_{-i})\prod_{j\in M\backslash \left\{i\right\}}\zeta_{j}(s_{j},a_{j})\right],$$

where $\prod_{j\in M\backslash \left\{i\right\}}\zeta_{j}(s_{j},a_{j})$ represents the joint probability of $SU_{i}$’s competing SUs choosing actions $a_{-i}$ in their respective states. From Equations (20) and (21), in multi-agent Q-learning, the agent needs not only its own transmission strategy but also the complete information of competing SUs’ strategies $\zeta_{j}\left(j\in M\backslash \left\{i\right\}\right)$ to update the Q-value of SU $n_{i}$. However, it is not always practical to observe other SUs’ private information in multi-hop CRN with finite cooperation. Therefore, designing a quasi-cooperative multi-agent learning scheme, which only needs private strategy and information exchange with its previous nodes, is challenging.

### 4.2. Equal Reward Time-Slots Based Conjectural Multi-Agent Q-Learning

Multi-agent learning strategy is more practical for solving the joint design problem in multi-hop CRN. The main drawback of establishing a multi-agent learning framework is the demand for complete information of competing SUs. Due to high communication overhead and topology complexity, it is impractical for SU nodes to cooperate with competing SUs and share their private information in multi-hop CRN. To resolve this contradiction, a conjecture-based multi-agent learning scheme with quasi-cooperative scenario is proposed, where each SU node conjectures other SUs’ behavior strategies without full coordination among agents.

Specifically, from Equations (20) and (21), the mixed-strategies for other competing SUs is defined as $\varphi_{i}^{t}(s_{i},a_{-i})=\prod_{j\in M\backslash \left\{i\right\}}\zeta_{j}^{t}(s_{j},a_{j})$, which represents the joint probability that competing SUs perform strategy vector $\zeta_{-i}={\left\{\zeta_{j}^{t}(s_{j},a_{j})\right\}}_{j\in M\backslash \left\{i\right\}}$ at time slot *t*. In other words, estimating $\varphi_{i}^{t}(s_{i},a_{-i})$ becomes the key challenge when applying the multi-agent Q-learning framework. To combat this, the conjecture belief ${\tilde{\varphi }}_{i}^{t}(s_{i},a_{-i})$ is introduced to approximate $\varphi_{i}^{t}(s_{i},a_{-i})$, and the ERT-CMQL is proposed to asymptotically determine ${\tilde{\varphi }}_{i}^{t}(s_{i},a_{-i})$ without complete network information. The probability that the agent chooses $a_{i}$ in state $s_{i}$ while other competing SUs execute action vector $a_{-i}$ is given by:

(22) $$\delta_{i}=\zeta_{i}^{t}(s_{i},a_{i})\cdot \varphi_{i}^{t}(s_{i},a_{-i}).$$

$SU_{i}$ receives expected reward $R_{i}(s_{i},a_{i},a_{-i})$ when the agent of $SU_{i}$ performs action $a_{i}$, while other nodes select action vector $a_{-i}$ in state $s_{i}$. That is, the probability that $SU_{i}$ acquires $R_{i}(s_{i},a_{i},a_{-i})$ is $\delta_{i}$. n is the number of time steps between any two moments in which $SU_{i}$ achieves the same return $R_{i}(s_{i},a_{i},a_{-i})$. Each n is independent of the others and follows the same distribution of $\delta_{i}$. The average value of n is denoted as $\bar{n}$, which can be obtained via the private information from historical observation. Then we have the approximate equation $\delta_{i}\approx 1/\left(1+\bar{n}\right)$ [15], i.e., $\zeta_{i}^{t}(s_{i},a_{i})\cdot \varphi_{i}^{t}(s_{i},a_{-i})\approx 1/\left(1+\bar{n}\right)$. Since every SU knows its own transmission strategy $\zeta_{i}^{t}(s_{i},a_{i})$, the agent can estimate $\varphi_{i}^{t}(s_{i},a_{-i})$ via:

(23) $${\tilde{\varphi }}_{i}^{t}(s_{i},a_{-i})=\frac{1}{\left(1+\bar{n})\cdot \zeta_{i}^{t}(s_{i},a_{i}\right)}.$$

After obtaining the expression of ${\tilde{\varphi }}_{i}^{t}(s_{i},a_{-i})$ using local information shown in Equation (23), the updating rule of the conjecture belief is explored. In quasi-cooperative learning scenarios, agents update their conjecture belief based on new observations. Since n is a stationary stochastic process in the time dimension, its mean value $\bar{n}$ is a constant. Specifically, the quotient of the conjecture belief at time slot *t* − 1 and *t* can be calculated as:

(24) $$\begin{array}{cc} \frac{{\tilde{\varphi }}_{i}^{t}(s_{i},a_{-i})}{{\tilde{\varphi }}_{i}^{t-1}(s_{i},a_{-i})} & =\frac{1}{\left(1+\bar{n})\cdot \zeta_{i}^{t}(s_{i},a_{i}\right)}/\left[\frac{1}{\left(1+\bar{n})\cdot \zeta_{i}^{t-1}(s_{i},a_{i}\right)}\right]\\ & =\frac{\zeta_{i}^{t-1}(s_{i},a_{i})}{\zeta_{i}^{t}(s_{i},a_{i})} \end{array}$$

Then, the conjecture belief is updated as follows:

(25) $${\tilde{\varphi }}_{i}^{t}(s_{i},a_{-i})={\tilde{\varphi }}_{i}^{t-1}(s_{i},a_{-i})\cdot \frac{\zeta_{i}^{t-1}(s_{i},a_{i})}{\zeta_{i}^{t}(s_{i},a_{i})}.$$

Since $\varphi_{i}^{t}(s_{i},a_{-i})=\prod_{j\in M\backslash \left\{i\right\}}\zeta_{j}^{t}(s_{j},a_{j})\approx {\tilde{\varphi }}_{i}^{t}(s_{i},a_{-i})$, the updating rule in Equation (20) can be rewritten as:

(26) $$Q_{i}^{t+1}(s_{i},a_{i})=(1-\alpha )Q_{i}^{t}(s_{i},a_{i})+\alpha \left[\sum_{(a_{i},a_{-i})\in A}R_{i}(s_{i},a_{i},a_{-i}){\tilde{\varphi }}_{i}^{t}(s_{i},a_{-i})+\beta \underset{b_{i}\in A_{i}}{\max}Q_{i}^{t}({s^{\prime }}_{i},b_{i})\right].$$

Equation (26) shows that every SU node only uses private strategy and limited information exchange with its previous nodes to update its Q-value. $SU_{i}$ conjectures the mixed-strategies for other competing SUs on the basis of their variations in response to their own strategy.

However, strong correlations exist between $\zeta_{i}^{t}(s_{i},a_{i})$ and $\zeta_{i}^{t-1}(s_{i},a_{i})$, which may cause the parameters to easily stick in a poor local optimum and then make $\zeta_{i}^{t-1}(s_{i},a_{i})/\zeta_{i}^{t}(s_{i},a_{i})$ close to 1 infinitely. Since the updating rule of ${\tilde{\varphi }}_{i}^{t}(s_{i},a_{-i})$ is fractional which has a strong reliance on $\zeta_{i}^{t-1}(s_{i},a_{i})/\zeta_{i}^{t}(s_{i},a_{i})$, the conjecture belief is also inclined to fall into the local optimal solution. To avoid the shortage and further improve the system performance, experience replay is applied to the conjecture based multi-agent learning scheme. From long-term-observations, $\bar{n}$ is a constant value due to the time stationarity of n. So the probability that $SU_{i}$ receives an expected reward $R_{i}(s_{i},a_{i},a_{-i})$ (i.e., agents perform action vector $\left(a_{i},a_{-i}\right)$ in state $s_{i}$) is approximately equal to the reciprocal of mean time interval regardless of time step, that is:

(27) $$\zeta_{i}^{t}(s_{i},a_{i})\cdot \varphi_{i}^{t}(s_{i},a_{-i})\approx \frac{1}{1+\bar{n}}\approx \zeta_{i}^{v}(s_{i},a_{i})\cdot \varphi_{i}^{v}(s_{i},a_{-i}),$$

where *t* and *v* represent any two time slots. Thus we have $\zeta_{i}^{t}(s_{i},a_{i})\cdot {\tilde{\varphi }}_{i}^{t}(s_{i},a_{-i})=\zeta_{i}^{v}(s_{i},a_{i})\cdot {\tilde{\varphi }}_{i}^{v}(s_{i},a_{-i})$. $\zeta_{i}(s_{i},a_{i})$ and ${\tilde{\varphi }}_{i}(s_{i},a_{-i})$ at each time step are stored as the agent’s experience at each time slot, and pooled over many episodes into a replay memory [27]. During learning, we randomly sample the experience ${\tilde{\varphi }}_{i}^{k}(s_{i},a_{-i})$ and $\zeta_{i}^{k}(s_{i},a_{i})$ from memory pool to update the conjecture. Then the update of conjecture belief at time step *t* is given by:

(28) $${\tilde{\varphi }}_{i}^{t}(s_{i},a_{-i})={\tilde{\varphi }}_{i}^{v}(s_{i},a_{-i})\cdot \frac{\zeta_{i}^{v}(s_{i},a_{i})}{\zeta_{i}^{t}(s_{i},a_{i})}.$$

This approach has several advantages over consecutive updating rule in Equation (25). First, each time-step of the strategy is potentially used in the update of conjecture, which improves data efficiency instead of updating directly from consecutive samples. Second, strong correlations between $\zeta_{i}^{t}(s_{i},a_{i})$ and $\zeta_{i}^{t-1}(s_{i},a_{i})$ may result in a local optimal. Randomizing the samples breaks these correlations and reduces the variance in the updates.

The details of ERT-CMQL are obtained as described in Algorithm 1.

**Algorithm 1** Equal Reward Time-Slots Based Conjectural Multi-Agent Q-Learning1: **Initialize:**2:    Set t=0 and memory size N.3:    **For** each $SU_{i}$
**Do**4:        **For** each $s_{i}\in S_{i},\;a_{i}\in A_{i}$
**Do**5:          Initialize transmission strategy $\zeta_{i}^{t}(s_{i},a_{i})$, conjecture belief ${\tilde{\varphi }}_{i}^{t}(s_{i},a_{-i})$,            Q-value $Q_{i}^{t}(s_{i},a_{i})$, and replay memory $D=\left\{{\tilde{\varphi }}_{i}^{t}(s_{i},a_{-i}),\;\zeta_{i}^{t}(s_{i},a_{i})\right\}$.6:       **End For**7:    **End For**8: **Repeated Learning:**9:    **For** each $SU_{i}$
**Do**10:      **For**
eposide=1, M
**do**11:         Initialize state $s_{i}^{1}$.12:         **Loop**13:            Select action $a_{i}^{t}$ according to the strategy $\zeta_{i}^{t}(s_{i},a_{i})$.14:            Execute action $a_{i}^{t}$, and obtain strategies of previous nodes $\zeta_{k}(s_{k},\;a_{k})$               and the SINR indicator $\rho_{i}$.15:            Observe reward $R_{i}^{t}(s_{i},a_{i},a_{-i})$ and state $s_{i}^{t+1}$ according to (14) and (16).16:            Update $Q_{i}^{t+1}(s_{i},a_{i})$ based on ${\tilde{\varphi }}_{i}^{t}(s_{i},a_{-i})$ according to (26).17:            Update the strategy ${\left\{\zeta_{i}^{t+1}(s_{i},a_{i})\right\}}_{a_{i}\in A_{i}}$ according to (19).18:            Sample experience ${\tilde{\varphi }}_{i}^{v}(s_{i},a_{-i})$ and $\zeta_{i}^{v}(s_{i},a_{i})$ from D.19:            Update the conjecture belief ${\tilde{\varphi }}_{i}^{t+1}(s_{i},a_{-i})$ according to (28).20:            Store ${\tilde{\varphi }}_{i}^{t+1}(s_{i},a_{-i})$ and $\zeta_{i}^{t+1}(s_{i},a_{i})$ in D.21:            $s_{i}=s_{i}^{t+1}$22:            t=t+123:         **Until**
$s_{i}$ is the terminal state24:      **End For**25:   **End For**

### 4.3. Analysis of ERT-CMQL

Littleman [28] provided the convergence proof of the standard Q-learning. Based on the theory, the convergence of ERT-CMQL is investigated in this section.

**Lemma.**  Suppose there is a mapping P:Q→Q, and Q denotes the set of all SUs’ Q functions. The updating rule $Q^{t+1}=(1-\alpha )Q^{t}+\alpha \cdot P\left(Q^{t}\right)$ converges to $Q^{*}$ with probability of 1, if:

(1) $Q^{*}=E\left[P\left(Q^{*}\right)\right]$
(2) A number 0<σ<1 exists such that $\left\|P\left(Q^{t}\right)-P\left(Q^{*}\right)\right\|\leq \sigma \left\|Q^{t}-Q^{*}\right\|$ for all $Q^{t}\in Q$.

To apply the lemma in the convergence proof of our proposed ERT-CMQL, the definition is given as follows:

**Definition.**  *Let $Q^{t}=\left(Q_{1}^{t},\ldots ,Q_{M}^{t}\right)$, where $Q_{i}^{t}\in Q_{i}$ for i∈M, and $Q=\prod_{i\in M}Q_{i}$. Then the mapping P:Q→Q is defined as $P\left(Q^{t}\right)=\left[P\left(Q_{1}^{t}\right),\ldots ,P\left(Q_{M}^{t}\right)\right]$, where:*

(29) $$P\left(Q_{i}^{t}(s_{i},a_{i})\right)=\sum_{(a_{i},a_{-i})\in A}R_{i}(s_{i},a_{i},a_{-i}){\tilde{\varphi }}_{i}^{t}(s_{i},a_{-i})+\beta \underset{b_{i}\in A_{i}}{\max}Q_{i}^{t}({s^{\prime }}_{i},b_{i}).$$

*In addition, for any $Q,\;Q^{\prime }\in Q$, the definition of the distance between Q-values is given as:*

(30) $$\left\|Q-Q^{\prime }\right\|=\underset{i\in M}{\max}\;\underset{s_{i}\in S_{i}}{\max}\;\underset{a_{i}\in A_{i}}{\max}\left|Q_{i}(s_{i},a_{i})-{Q_{i}}^{\prime }(s_{i},a_{i})\right|.$$

Firstly, we prove the first condition in Lemma 1 for ERT-CMQL.

**Proposition** **1.** $Q^{*}$ *is equal to the expectation of its map $P\left(Q^{*}\right)$, i.e., $Q^{*}=E\left[P\left(Q^{*}\right)\right]$, where $Q^{*}=\left(Q_{1}^{*},\ldots ,Q_{M}^{*}\right)$.*

**Proof.**  According to the Bellman’s optimality equation [29], we have the following expression:

(31) $$Q_{i}^{*}(s_{i},a_{i})=E\left[R_{i}(s_{i},a_{i},\zeta_{-i}^{*})\right]+\beta \sum_{{s^{\prime }}_{i}\in S_{i}}P_{s_{i},{s^{\prime }}_{i}}(a_{i},\zeta_{-i}^{*})\underset{b_{i}\in A_{i}}{\max}Q_{i}^{*}({s^{\prime }}_{i},b_{i}),$$

Since the reward $R_{i}(s_{i},a_{i},\zeta_{-i}^{*})$ is irrelevant to ${s^{\prime }}_{i}$, then Equation (31) can be modified as:

(32) $$\begin{array}{cc} Q_{i}^{*}(s_{i},a_{i}) & =\sum_{{s^{\prime }}_{i}\in S_{i}}P_{s_{i},{s^{\prime }}_{i}}(a_{i},\zeta_{-i}^{*})\left\{E\left[R_{i}(s_{i},a_{i},\zeta_{-i}^{*})\right]+\beta \underset{b_{i}\in A_{i}}{\max}Q_{i}^{*}({s^{\prime }}_{i},b_{i})\right\}\\ & =\sum_{{s^{\prime }}_{i}\in S_{i}}P_{s_{i},{s^{\prime }}_{i}}(a_{i},\zeta_{-i}^{*})\left\{\sum_{(a_{i},a_{-i})\in A}R_{i}(s_{i},a_{i},a_{-i})\prod_{j\in M\backslash \left\{i\right\}}\zeta_{j}(s_{j},a_{j})+\beta \underset{b_{i}\in A_{i}}{\max}Q_{i}^{*}({s^{\prime }}_{i},b_{i})\right\} \end{array}$$

Based on the previous analysis in Section 4.2, we have $\varphi_{i}^{t}(s_{i},a_{-i})=\prod_{j\in M\backslash \left\{i\right\}}\zeta_{j}^{t}(s_{j},a_{j})$. Then we prove that $Q_{i}^{*}(s_{i},a_{i})=E\left[P\left(Q_{i}^{*}(s_{i},a_{i})\right)\right]$.  □

**Proposition** **2.** *There is a number 0<σ<1 such that $\left\|P\left(Q^{t}\right)-P\left(Q^{*}\right)\right\|\leq \sigma \left\|Q^{t}-Q^{*}\right\|$.*

**Proof.**  In accordance with the definition of the distance between Q-values, we have:

(33) $$\begin{array}{ccc} \left\|P\left(Q\right)-P\left(Q^{\prime }\right)\right\| & = & \underset{i\in M}{\max}\;\underset{s_{i}\in S_{i}}{\max}\;\underset{a_{i}\in A_{i}}{\max}\left|P\left(Q_{i}(s_{i},a_{i})\right)-P\left({Q_{i}}^{\prime }(s_{i},a_{i})\right)\right|\\ & = & \underset{i\in M}{\max}\;\underset{s_{i}\in S_{i}}{\max}\;\underset{a_{i}\in A_{i}}{\max}\left|\begin{array}{l} \sum_{(a_{i},a_{-i})\in A}R_{i}(s_{i},a_{i},a_{-i})\left[{\tilde{\varphi }}_{i}(s_{i},a_{-i})-{{\tilde{\varphi }}^{\prime }}_{i}(s_{i},a_{-i})\right]\\ +\beta \left[\underset{b_{i}\in A_{i}}{\max}Q_{i}({s^{\prime }}_{i},b_{i})-\underset{b_{i}\in A_{i}}{\max}{Q^{\prime }}_{i}({s^{\prime }}_{i},b_{i})\right] \end{array}\right|\\ & \leq & \underset{i\in M}{\max}\;\underset{s_{i}\in S_{i}}{\max}\;\underset{a_{i}\in A_{i}}{\max}\left|\sum_{(a_{i},a_{-i})\in A}R_{i}(s_{i},a_{i},a_{-i})\left[{\tilde{\varphi }}_{i}(s_{i},a_{-i})-{{\tilde{\varphi }}^{\prime }}_{i}(s_{i},a_{-i})\right]\right|\\ & & +\underset{i\in M}{\max}\;\underset{s_{i}\in S_{i}}{\max}\;\underset{a_{i}\in A_{i}}{\max}\beta \left|\underset{b_{i}\in A_{i}}{\max}Q_{i}({s^{\prime }}_{i},b_{i})-\underset{b_{i}\in A_{i}}{\max}{Q^{\prime }}_{i}({s^{\prime }}_{i},b_{i})\right|\\ & \leq & \underset{i\in M}{\max}\;\underset{s_{i}\in S_{i}}{\max}\;\underset{a_{i}\in A_{i}}{\max}\left|\sum_{(a_{i},a_{-i})\in A}R_{i}(s_{i},a_{i},a_{-i})\left[{\tilde{\varphi }}_{i}(s_{i},a_{-i})-{{\tilde{\varphi }}^{\prime }}_{i}(s_{i},a_{-i})\right]\right|\\ & & +\underset{i\in M}{\max}\;\underset{s_{i}\in S_{i}}{\max}\;\underset{a_{i}\in A_{i}}{\max}\left[\underset{b_{i}\in A_{i}}{\max}\beta \left|Q_{i}({s^{\prime }}_{i},b_{i})-{Q^{\prime }}_{i}({s^{\prime }}_{i},b_{i})\right|\right]\\ & = & \underset{i\in M}{\max}\;\underset{s_{i}\in S_{i}}{\max}\;\underset{a_{i}\in A_{i}}{\max}\left|\sum_{(a_{i},a_{-i})\in A}R_{i}(s_{i},a_{i},a_{-i})\left[{\tilde{\varphi }}_{i}(s_{i},a_{-i})-{{\tilde{\varphi }}^{\prime }}_{i}(s_{i},a_{-i})\right]\right|+\beta \left\|Q-Q^{\prime }\right\| \end{array}$$

where the second equation is derived from Equation (29), and the first inequality is obtained according to the transformation |A+B|≤|A|+|B|. It can be easily proven that |maxA−maxB|≤max|A−B|, so we can attain the second inequality. Since $a_{i}$ is unrelated to $Q_{i}({s^{\prime }}_{i},b_{i})-{Q^{\prime }}_{i}({s^{\prime }}_{i},b_{i})$, $\underset{i\in M}{\max}\;\underset{s_{i}\in S_{i}}{\max}\;\underset{a_{i}\in A_{i}}{\max}\left[\underset{b_{i}\in A_{i}}{\max}\beta \left|Q_{i}({s^{\prime }}_{i},b_{i})-{Q^{\prime }}_{i}({s^{\prime }}_{i},b_{i})\right|\right]$ can be rewritten as $\underset{i\in M}{\max}\;\underset{s_{i}\in S_{i}}{\max}\;\underset{b_{i}\in A_{i}}{\max}\beta \left|Q_{i}({s^{\prime }}_{i},b_{i})-{Q^{\prime }}_{i}({s^{\prime }}_{i},b_{i})\right|$ and the last equation is attained using Equation (30).

Next, we apply Equation (23) to the item $\sum_{(a_{i},a_{-i})\in A}R_{i}(s_{i},a_{i},a_{-i})\left[{\tilde{\varphi }}_{i}(s_{i},a_{-i})-{{\tilde{\varphi }}^{\prime }}_{i}(s_{i},a_{-i})\right]$, and the expression can be rewritten as:

(34) $$\begin{array}{cc} & \sum_{(a_{i},a_{-i})\in A}R_{i}(s_{i},a_{i},a_{-i})\left[{\tilde{\varphi }}_{i}(s_{i},a_{-i})-{{\tilde{\varphi }}^{\prime }}_{i}(s_{i},a_{-i})\right]\\ = & \sum_{(a_{i},a_{-i})\in A}R_{i}(s_{i},a_{i},a_{-i})\cdot \left[\frac{1}{\left(1+\bar{n})\zeta_{i}(s_{i},a_{i}\right)}-\frac{1}{\left(1+\bar{n}){\zeta^{\prime }}_{i}(s_{i},a_{i}\right)}\right]\\ = & \sum_{(a_{i},a_{-i})\in A}R_{i}(s_{i},a_{i},a_{-i})\cdot \left[\frac{{\zeta^{\prime }}_{i}(s_{i},a_{i})}{\left(1+\bar{n})\zeta_{i}(s_{i},a_{i}){\zeta^{\prime }}_{i}(s_{i},a_{i}\right)}-\frac{\zeta_{i}(s_{i},a_{i})}{\left(1+\bar{n})\zeta_{i}(s_{i},a_{i}){\zeta^{\prime }}_{i}(s_{i},a_{i}\right)}\right]\\ = & \sum_{(a_{i},a_{-i})\in A}R_{i}(s_{i},a_{i},a_{-i})\cdot \frac{{\zeta^{\prime }}_{i}(s_{i},a_{i})-\zeta_{i}(s_{i},a_{i})}{\left(1+\bar{n})\zeta_{i}(s_{i},a_{i}){\zeta^{\prime }}_{i}(s_{i},a_{i}\right)} \end{array}$$

where $R_{i}(s_{i},a_{i},a_{-i})$ is the reward when $SU_{i}$ selects action $a_{i}$ in state $s_{i}$, while other nodes select action vector $a_{-i}$, $\bar{n}$ is the average number of time steps between any two moments in which $SU_{i}$ achieves the same return $R_{i}(s_{i},a_{i},a_{-i})$, and $\zeta_{i}(s_{i},a_{i})$ and ${\zeta^{\prime }}_{i}(s_{i},a_{i})$ are the strategies of $SU_{i}$ in state $s_{i}$ at different time-step.

When η is sufficiently large, we have:

(35) $$e^{Q_{i}\left(s_{i},a_{i}\right)/\eta }=1+\frac{Q_{i}\left(s_{i},a_{i}\right)}{\eta }+ο\left({\left(\frac{Q_{i}\left(s_{i},a_{i}\right)}{\eta }\right)}^{2}\right)=1+\frac{Q_{i}\left(s_{i},a_{i}\right)}{\eta }+\omega \left(\frac{Q_{i}\left(s_{i},a_{i}\right)}{\eta }\right),$$

and:

(36) $$\sum_{b\in A_{i}}e^{Q_{i}\left(s_{i},b\right)/\eta }=\left|A_{i}\right|+\sum_{b\in A_{i}}\left[\frac{Q_{i}\left(s_{i},b\right)}{\eta }+\omega \left(\frac{Q_{i}\left(s_{i},b\right)}{\eta }\right)\right],$$

where $\omega \left(\frac{Q_{i}\left(s_{i},a_{i}\right)}{\eta }\right)$ is a polynomial of order $ο\left({\left(\frac{Q_{i}\left(s_{i},a_{i}\right)}{\eta }\right)}^{2}\right)$. By applying Equations (35) and (36) to Equation (19), it can be verified that:

(37) $$\zeta_{i}(s_{i},a_{i})=\frac{1}{\left|A_{i}\right|}+\frac{1}{\left|A_{i}\right|}\cdot \frac{Q_{i}\left(s_{i},a_{i}\right)}{\eta }+\varpi \left({\left\{\frac{Q_{i}\left(s_{i},b\right)}{\eta }\right\}}_{b}\right),$$

and:

(38) $${\zeta^{\prime }}_{i}(s_{i},a_{i})=\frac{1}{\left|A_{i}\right|}+\frac{1}{\left|A_{i}\right|}\cdot \frac{{Q^{\prime }}_{i}\left(s_{i},a_{i}\right)}{\eta }+\varpi \left({\left\{\frac{{Q^{\prime }}_{i}\left(s_{i},b\right)}{\eta }\right\}}_{b}\right),$$

where $\varpi \left({\left\{\frac{Q_{i}\left(s_{i},b\right)}{\eta }\right\}}_{b}\right)$ is a polynomial of order smaller than $ο\left({\left\{\frac{Q_{i}\left(s_{i},a_{i}\right)}{\eta }\right\}}_{b}\right)$.

Substituting Equations (37) and (38) into Equation (34), we have:

(39) $$\begin{array}{cc} & \sum_{(a_{i},a_{-i})\in A}R_{i}(s_{i},a_{i},a_{-i})\cdot \frac{{\zeta^{\prime }}_{i}(s_{i},a_{i})-\zeta_{i}(s_{i},a_{i})}{\left(1+\bar{n})\zeta_{i}(s_{i},a_{i}){\zeta^{\prime }}_{i}(s_{i},a_{i}\right)}\\ =- & \sum_{(a_{i},a_{-i})\in A}\frac{R_{i}(s_{i},a_{i},a_{-i})}{\left(1+\bar{n})\zeta_{i}(s_{i},a_{i}){\zeta^{\prime }}_{i}(s_{i},a_{i}\right)}\left(\begin{array}{l} \frac{1}{\eta \left|A_{i}\right|}\cdot \left[Q_{i}(s_{i},a_{i})-{Q^{\prime }}_{i}(s_{i},a_{i})\right]\\ +\varpi \left({\left\{\frac{Q_{i}\left(s_{i},b\right)}{\eta }\right\}}_{b}\right)-\varpi \left({\left\{\frac{{Q^{\prime }}_{i}\left(s_{i},b\right)}{\eta }\right\}}_{b}\right) \end{array}\right)\\ =- & \sum_{(a_{i},a_{-i})\in A}\frac{R_{i}(s_{i},a_{i},a_{-i})}{\eta (1+\bar{n})\zeta_{i}(s_{i},a_{i}){\zeta^{\prime }}_{i}(s_{i},a_{i})}\cdot \frac{1}{\left|A_{i}\right|}\cdot \left[Q_{i}(s_{i},a_{i})-{Q^{\prime }}_{i}(s_{i},a_{i})\right]+\varpi \left({\left\{\frac{Q_{i}\left(s_{i},b\right)}{\eta }\right\}}_{b}\right)-\varpi \left({\left\{\frac{{Q^{\prime }}_{i}\left(s_{i},b\right)}{\eta }\right\}}_{b}\right) \end{array}$$

A sufficiently large η can be taken so that:

(40) $$\left|\frac{R_{i}(s_{i},a_{i},a_{-i})}{\eta (1+\bar{n})\zeta_{i}(s_{i},a_{i}){\zeta^{\prime }}_{i}(s_{i},a_{i})}\right|\leq 1-\beta .$$

Then we have the following inequality:

(41) $$\left|\sum_{(a_{i},a_{-i})\in A}R_{i}(s_{i},a_{i},a_{-i})\left[{\tilde{\varphi }}_{i}(s_{i},a_{-i})-{{\tilde{\varphi }}^{\prime }}_{i}(s_{i},a_{-i})\right]\right|\leq \frac{1-\beta }{\left|A_{i}\right|}\cdot \left|Q_{i}(s_{i},a_{i})-{Q^{\prime }}_{i}(s_{i},a_{i})\right|,$$

which leads to:

(42) $$\begin{array}{cc} \left\|P\left(Q\right)-P\left(Q^{\prime }\right)\right\|\leq & \underset{i\in M}{\max}\;\underset{s_{i}\in S_{i}}{\max}\;\underset{a_{i}\in A_{i}}{\max}\frac{1-\beta }{\left|A_{i}\right|}\cdot \left|Q_{i}(s_{i},a_{i})-{Q^{\prime }}_{i}(s_{i},a_{i})\right|+\beta \left\|Q-Q^{\prime }\right\|\\ & \leq \frac{1-\beta }{\upsilon }\left\|Q-Q^{\prime }\right\|+\beta \left\|Q-Q^{\prime }\right\|=\frac{1-\beta +\beta \upsilon }{\upsilon }\left\|Q-Q^{\prime }\right\| \end{array}$$

where $\upsilon =\underset{i\in N}{\min}\left|A_{i}\right|>1$. Then we have υ⋅(1−β)>1−β, so that υ−βυ>1−β, which leads to $\frac{1-\beta +\beta \upsilon }{\upsilon }<1$. Consequently, condition (2) is satisfied in the Lemma, and ERT-CMQL is proven to converge if η is large enough for all agents.  □

## 5. Simulation Results

In this section, the performance of our quasi-cooperative multi-agent learning scheme is evaluated using an event-driven simulator coded in Python 3.5. The network model and learning framework are built based on the Python packages Networkx and Numpy, respectively. The results of the proposed ERT-CMAQL are compared with (1) Cooperative Multi-Agent Q-Learning (CMAQL) which is the ideal scheme and has complete information of the competing SUs; (2) Conjectural Multi-Agent Q-Learning without Experience Replay (CMAQL-ER); (3) Fixed Power- based Conjectural Multi-Agent Q-Learning (FP-CMAQL) proposed in [16] which transmits data with a fixed power level; (4) a single agent Q-learning scheme called Q-routing presented in [14] and (5) Prioritized Memories Deep Q-Network (PM-DQN) based joint design scheme proposed in our previous work [17]. 

In the multi-agent learning framework, we initialize the conjecture belief ${\tilde{\varphi }}_{i}^{0}(s_{i},a_{-i})=1$, the Q-value $Q_{i}^{0}(s_{i},a_{i})=0$, and the transmission strategy $\zeta_{i}^{0}(s_{i},a_{i})=1/\left|A_{i}\right|$ for each $s_{i}\in S_{i},\;a_{i}\in A_{i}$. Other system parameters are given in Table 2.

**Figure 2:**
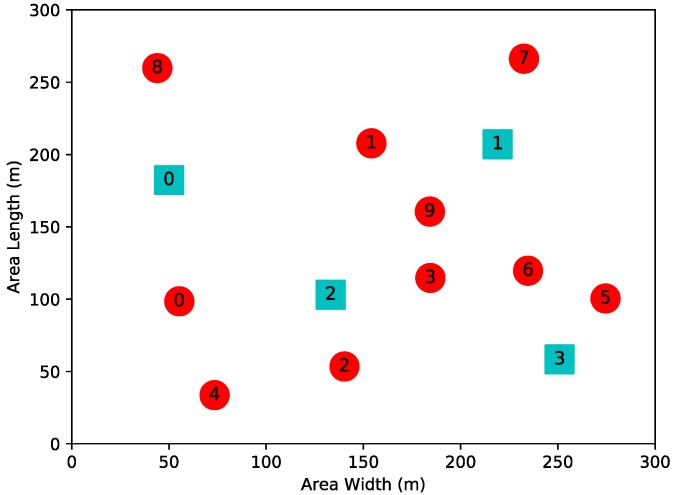
Network topology consisting of 10 SUs and 4 PUs.

To verify the performance of algorithms, a small CRN containing 10 SUs and 4 PUs is simulated at first. SUs in CRN are uniformly deployed in a 300 × 300 m region. In addition, the available transmitting power consists of five levels: {50, 100, …, 250 mW}. The network topology is shown in Figure 2.

**Figure 3:**
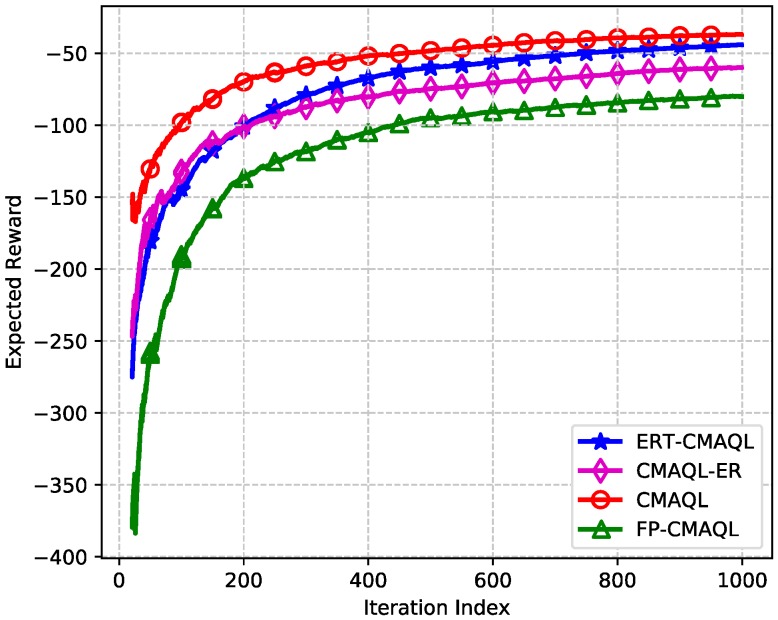
Expected reward of the SU 6 versus the iteration index.

**Figure 4:**
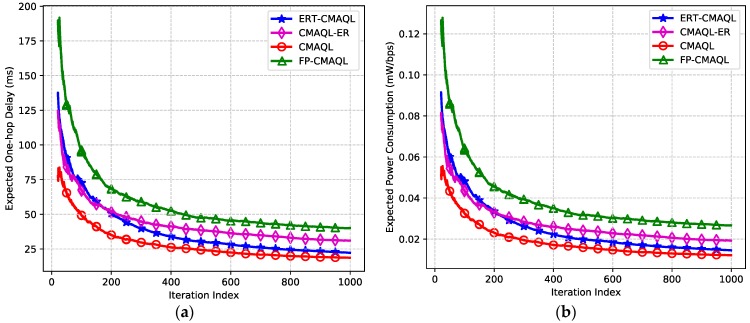
(**a**) Expected end-to-end delay and (**b**) expected power consumption ratio.

Without loss of generality, SU 6 is taken as an example. The single-node performance of SU 6 for different algorithms is shown in Figure 3 and Figure 4. Figure 3 illustrates the average reward of SU 6 versus the iteration index. The expected reward firstly rises and then stays almost steady for all schemes. Furthermore, we find that, when converged, CMAQL outperforms all other algorithms. The reward of ERT-CMAQL is slightly lower than CMAQL, followed by the CMAQL-ER scheme, and FP-CMAQL obtains the lowest reward. This occurs mainly because, in the CMAQL scheme, agents have true strategies of competing SUs through global information exchange. In ERT-CMAQL, each agent approximates mixed-strategies of other SUs via the conjecture belief that may be not sufficiently accurate. We can see that the reward of CMAQL-ER is slightly higher than that of ERT-CMAQL before 200 iterations, and afterward, that ERT-CMAQL is superior to CMAQL-ER. The reason for this is that at first few samples are stored in replay memory and the correlation is weak between the samples, so that experience replay is inefficient compared to the consecutive updating rule. At the later stage the advantage of experience replay is fully demonstrated when samples are abundant. In addition, FP-CMAQL obtains the lowest average reward, which illustrates the importance of power allocation.

The effectiveness properties of transmission latency and power efficiency are demonstrated in this part. In Figure 4a, single-hop latency declines in the beginning and flattens after about 700 iterations for all kinds of protocols. CMAQL achieves the lowest transmission latency, which is a little shorter than that of ERT-CMAQL. The expected delay of ERT-CMAQL is about 32% lower than CMAQL-ER, which benefits from experience replay to avoid a poor local minimum and enhace data efficiency. The transmission delay of FP-CMAQL is much longer than the other three schemes because it fails to adjust the transmission power with channel status causing larger overall latency. Figure 4b shows the average power consumption versus iteration index. The PCR of the four algorithms, in increasing order, is as follows: CMAQL, ERT-CMAQL, CMAQL-ER and FP-CMAQL. The reason for this is the same as in Figure 4a. Consequently, the results illustrate that the energy efficiency of proposed ERT-CMAQL is close to the optimum scheme, and holds a clear advantage over other algorithms.

**Figure 5:**
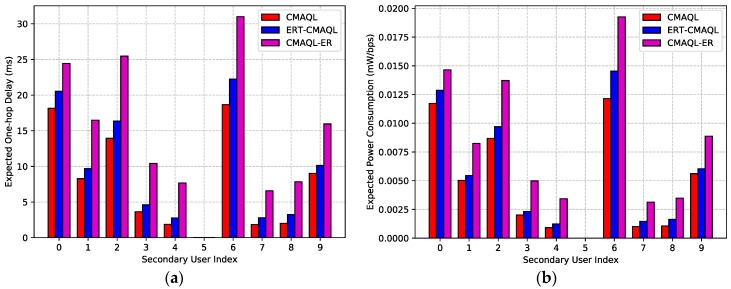
(**a**) Expected single-hop delay and (**b**) expected power consumption.

Figure 5a shows the expected one-hop delay of different SU nodes for CMAQL, ERT-CMAQL, and CMAQL-ER schemes. The transmission delay of CMAQL is the lowest compared with the other two algorithms, the latency of ERT-CMAQL is slightly higher than that of CMAQL, and CMAQL-ER achieves the longest one-hop delay for all SU nodes in the network. This illustrates the effect of experience replay, which makes the performance of ERT-CMAQL close to the optimum, demonstrating a clear advantage over the schemes applying the consecutive updating rule. 

**Figure 6:**
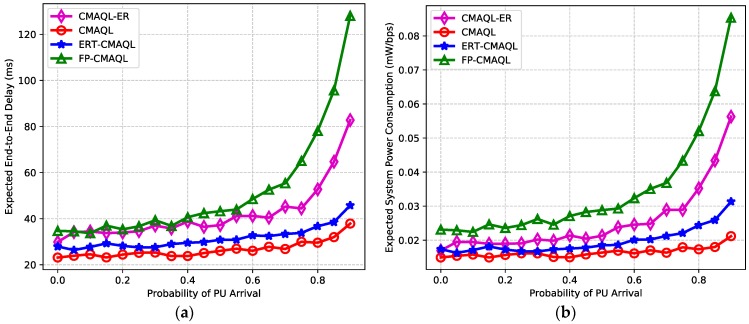
(**a**) Expected end-to-end delay and (**b**) expected system power consumption.

In addition, we find that SU 6 achieves the longest expected one-hop delay of all SUs, while the transmission latency of SU 4 and SU 7 is relatively low on average. This is due to SU 6 being the closest SU to the destination node so that data flows pass through it with higher probability. The locations of SU 4 and SU 7 are relatively isolated, so the arrival packets are scarce. SU 5 is the destination node and no packet is transmitted forward so that its transmission delay is 0. Comparison of PCR for the three kinds of protocols varying in the SU index is shown in Figure 5b. We can find that the PCR of ERT-CMAQL is close to CMAQL for all SUs. CMAQL-ER achieves the highest PCR of the three schemes for all SU nodes, which consumes 46% more power per throughput on average than ERT-CMAQL. The reason for this is similar to the reason for the results in Figure 5a. Therefore, the proposed ERT-CMAQL achieves relatively low transmission latency and power consumption close to the optimum for every SU node in the network. Figure 6 illustrates the effect of PU arrival probability on expected end-to-end delay and system PCR. As shown in Figure 6a, it is found that the transmission latency of the four algorithms grows as the PU arrival probability increases. This is because the larger PU arrival probability results in more interruption and transmission failure, which causes longer delays for data retransmission. CMAQL achieves the lowest transmission delay, followed by ERT-CMAQL. The latency of CMAQL-ER is higher than that of ERT-CMAQL, and FP-CMAQL has the longest expected end-to-end latency. This demonstrates the advantage of our proposed ERT-CMAQL, which produces performance closest to the ideal value. Furthermore, the transmission latency values of the four algorithms are relatively close when PU arrival probability is low. However, they differ considerably as PU arrival probability increases. When the probability of PU arrival is low, there is little conflict between PUs and SUs so the four algorithms achieve almost the same latency. Since CMAQL and ERT-CMAQL can better avoid conflicts with PU, CMAQL and ERT-CMAQL are capable of maintaining relatively low latency when PU arrival probability increases. However, in CMAQL-ER and FP-CMAQL, the data transmission of SUs is often interrupted by PU arrival so the transmission latency is high. The effect of PU arrival probability on PCR shown in Figure 6b has a similar trend to Figure 6a, which will not be detailed here.

**Figure 7:**
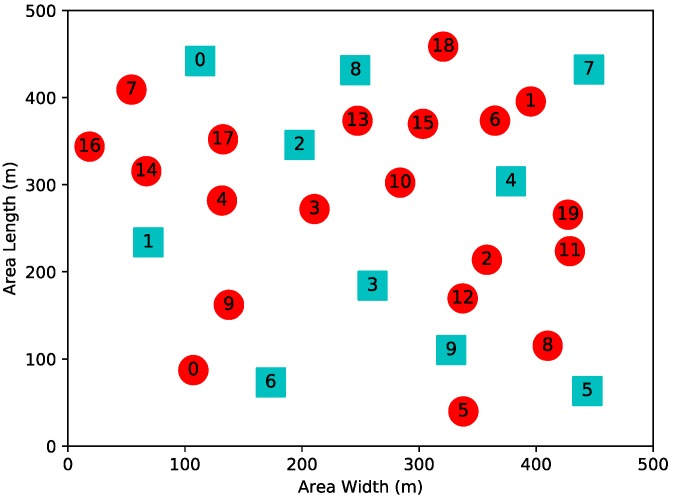
Network topology consisting of 20 SUs and 10 PUs.

Next, for a more general case, a networking scenario comprising 20 SUs and 10 PUs uniformly deployed in a 500 × 500 m area is considered. The available transmit power contains ten levels: {50, 100, …, 500 mW}. The network topology of the second experiental scenario is shown in Figure 7.

**Figure 8:**
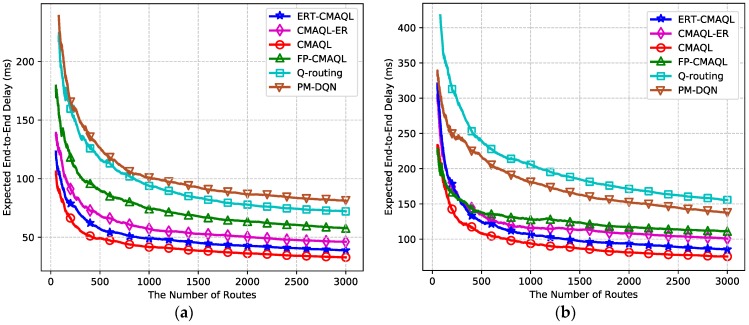
Expected end-to-end delay versus the number of routes: (**a**) 10 SU nodes, 4 PU channels and (**b**) 20 SU nodes, 10 PU channels.

The comparison of system performance for different kinds of experimental environments is illustrated in this section. Figure 8 depicts the expected end-to-end latency of six algorithms in networks with 10 SUs and 20 SUs. It can be seen that with increasing number of routes, the end-to-end delay sharply declines and then remains steady for all algorithms in both networking scenarios. When converged, CMAQL achieves the lowest end-to-end delay due to the complete information, which helps agents make more accurate and comprehensive decisions. Given the conjecture belief and experience replay, the total transmission delay of ERT-CMAQL is close to CMAQL. CMAQL-ER, with its consecutive updating rule, consumes more time transmiting packets from the source to the destination than ERT-CMAQL, followed by FP-CMAQL. The transmission latency of the two single-agent schemes is particularly larger because, in these two schemes, all the information and computations are processed by a separate agent, which is inherently less efficient than multi-agent schemes. PM-DQN produces a longer end-to-end delay than Q-routing in the network with 10 SUs, but its performance is superior to Q-routing in a large network. This illustrates the advantage of PM-DQN in the networking scenarios with large state space.

By comparing the performance of the two networking scenarios, we can find that the end-to-end latency of all protocols in the network with 20 SUs is relatively longer than in the small-scale network, and the latency of single-agent schemes increases more apparently than in other algorithms. The reason for this is that the links of the route increase with increasing SUs in the network, so that the accumulated single-hop latency along the route, i.e., the end-to-end delay, grows as well. Single-agent schemes are more sensitive to the number of SUs, which leads to longer latency in total. Furthermore, it is observed that the convergence speed of multi-agent schemes remains almost the same in both networking scenarios. However, Q-routing and PM-DQN converge at around 1700 routes in the first experiment, and nearly 3000 routes in the second. From the theoretical analysis, we find that multi-agent learning schemes are not affected by network scale because each SU equips an agent and follows the same learning rule. The calculation load rises as the number of SUs grows, which has heavy impact on single-agent schemes with only one agent in the network.

**Figure 9:**
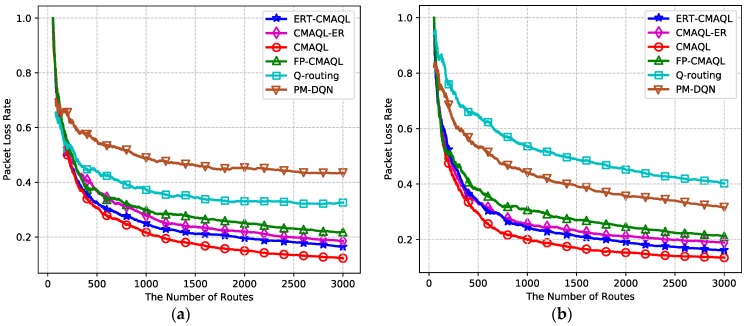
Packet loss ratio versus the number of routes: (**a**) 10 SU nodes, 4 PU channels and (**b**) 20 SU nodes, 10 PU channels.

We further investigate the packet loss ratio (PLR) of the six protocols for different networking scenarios in Figure 9. In both experimental environments, CMAQL has the lowest PLR, followed by the proposed ERT-CMAQL. The PLR of Q-routing and PM-DQN is obviously higher than other multi-agent learning schemes, which illustrates the reliability of using multiple agents. Comparing Figure 9a,b, we find that the PLR of Q-routing is larger than PM-DQN in the first network, whereas PM-DQN is more robust than Q-routing in large-scale networks. This is because PM-DQN has higher efficiency in networks with large state space due to the capability of the neural network. In addition, the PLR of all multi-agent schemes remains almost the same, which demonstrates that the routing reliability is not affected by network scale for multi-agent learning algorithms. The reason for this finding is that, in multi-agent collaboration schemes, every SU node equips an agent regardless of the network size, which improves the robustness as the number of SUs increases.

## 6. Conclusions

In this paper, we developed a quasi-cooperative multi-agent learning scheme for multi-hop CRN called ERT-CMAQL. The simulation results show that ERT-CMAQL reduces the expected end-to-end latency, guarantees the robustness of routing and achieves higher power efficiency compared to traditional learning algorithms, and its performance is close to CMAQL using complete information. In this paper, every SU agent learns the information of topology and channel statistics by itself. However, self-learning faces two crucial challenges: it requires a large number of interactions between agents and environment, which takes considerable time, and some energy-constraint applications cannot afford to the large power expenditure due to the trial and error manner of RL. Unlike general learning strategies, apprenticeship learning allows newly-jointed SUs to learn from the expert nodes with mature experience, which makes the joint optimization algorithm converge faster and achieve better performance. Our future work will aim to adopt the apprenticeship learning strategy to accelerate the learning process in CRN.

## Figures and Tables

**Table 1:** List of Acronyms.

Abbreviation	Full Name
SUs	Secondary Users
PUs	Primary Users
CRN	Cognitive Radio Networks
QoS	Quality of Service
CR	Cognitive Radio
SG	Stochastic Game
DTC	Data Transmission Channel
CCC	Common Control Channel
PDF	Probability Density Function
TL	Transmission Latency
PCR	Power Consumption Ratio
AWGN	Additive White Gaussian Noise
SINR	Signal-to-Interference plus Noise Ratio
RL	Reinforcement Learning
MSNE	Mixed-Strategy Nash Equilibrium
PLR	Packet Loss Ratio

**Table 2:** System Parameters.

Parameters	Values
Link Gain	$h=\varepsilon G{\left(r/r_{0}\right)}^{-m}，\;\mathrm{for}\;r>r_{0}$ [15]
Available Spectrum	56 MHz − 62 MHz
Bandwidth, B	1 MHz
AWGN power, ϑ	$10^{-7}\;\mathrm{mW}$
PU-to-SU interference, $\phi_{ijc}^{PU}$	$\phi_{ijc}^{PU}\sim \left[10^{-7},\;10^{-6}\right]\;\mathrm{mW}$
Packet Size, $R_{packet}$	$2\times 10^{5}\;\mathrm{bit}$
Mean of PU Departure Rate, μ	0.1
Deviation of PU Departure Rate, σ	0.05
SINR threshold, $\gamma_{th}$	60 dBm
Outage probability, $P_{ij}^{out}$	$P_{ij}^{out}\sim N\left(0.1,\;0.05\right)$
Data flow arrival rate, $\lambda_{f}$	0.4
Data flow service rate, $\mu_{f}$	0.6
Maximal transmission delay, $\kappa_{th}$	200 ms
Discount factor, β	0.9
Learning rate, α	0.1
Time step size, *t*	500 ms
Temperature, η	0.005

## References

[1] Hossain E., Niyato D., Kim D.I. (2015). Evolution and Future Trends of Research in Cognitive Radio: A Contemporary Survey. Wirel. Commun. Mob. Comput..

[2] Ahmad A., Ahmad S., Rehmani M.H., Hassan N.U. (2015). A Survey on Radio Resource Allocation in Cognitive Radio Sensor Networks. IEEE Commun. Surv. Tutor..

[3] Ma Y., Zhou L., Liu K. (2013). A Subcarrier-Pair based Resource Allocation Scheme Using Proportional Fairness for Cooperative OFDM-based Cognitive Radio Networks. Sensors.

[4] Huang J., Zeng X., Jian X., Tan X., Zhang Q. (2017). Opportunistic Capacity-based Resource Allocation for Chunk-based Multi-carrier Cognitive Radio Sensor Networks. Sensors.

[5] Zareei M., Islam A.K.M.M., Baharun S., Vargasrosales C., Azpilicueta L., Mansoor N. (2017). Medium Access Control Protocols for Cognitive Radio Ad Hoc Networks: A Survey. Sensors.

[6] Ruby E.D.K., Saranya N., Santhkumar W.E. (2014). A survey on distributed channel selection technique using surf algorithm for information transfer in multi-hop cognitive radio networks. Int. Conf. Comput. Sci. Comput. Intell..

[7] Liu Y., Cai L.X., Shen X.S. (2012). Spectrum-Aware Opportunistic Routing in Multi-Hop Cognitive Radio Networks. IEEE J. Sel. Areas Commun..

[8] Ding L., Melodia T., Batalama S.N., Matyjas J.D. Distributed Routing, Relay Selection, and Spectrum Allocation in Cognitive and Cooperative Ad Hoc Networks. Proceedings of the 7th Annual IEEE Communications Society Conference on Sensor, Mesh and Ad Hoc Communications and Networks (SECON).

[9] Lai L., Wang J., Huang A., Shan H. Routing and Resource Allocation with Collision Constraint in Multi-Hop Cognitive Radio Networks. Proceedings of the GLOBECOM Workshops.

[10] Amini R.M., Dziong Z. (2014). An Economic Framework for Routing and Channel Allocation in Cognitive Wireless Mesh Networks. IEEE Trans. Netw. Serv. Manag..

[11] Royer E.M., Toh C.K. (2002). A Review of Current Routing Protocols for Ad Hoc Mobile Wireless Networks. IEEE Pers. Commun..

[12] Bkassiny M., Li Y., Jayaweera S.K. (2013). A Survey on Machine-Learning Techniques in Cognitive Radios. IEEE Commun. Surv. Tutor..

[13] Raj V., Dias I., Tholeti T., Kalyani S. (2018). Spectrum Access in Cognitive Radio Using A Two Stage Reinforcement Learning Approach. IEEE J. Sel. Top. Signal Process..

[14] Al-Rawi H.A.A., Yau K.L.A., Mohamad H. A Reinforcement Learning-based Routing Scheme for Cognitive Radio Ad Hoc Networks. Proceedings of the 7th IFIP Wireless and Mobile Networking Conference (WMNC).

[15] Chen X., Zhao Z., Zhang H. (2013). Stochastic Power Adaptation with Multiagent Reinforcement Learning for Cognitive Wireless Mesh Networks. IEEE Trans. Mobile Comput..

[16] Pourpeighambar B., Dehghan M., Sabaei M. (2017). Non-Cooperative Reinforcement Learning based Routing in Cognitive Radio Networks. Comput. Commun..

[17] Du Y., Zhang F., Xue L. (2018). A Kind of Joint Routing and Resource Allocation Scheme based on Prioritized Memories-Deep Q Network for Cognitive Radio Ad Hoc Networks. Sensors.

[18] El-Sherif A.A., Mohamed A., Hu Y.C. Joint Routing and Resource Allocation for Delay Sensitive Traffic in Cognitive Mesh Networks. Proceedings of the IEEE Globecom Workshops.

[19] Singh K., Moh S. (2017). An Energy-Efficient and Robust Multipath Routing Protocol for Cognitive Radio Ad Hoc Networks. Sensors.

[20] Al-Rawi H.A.A., Yau K.L.A. Route Selection for Minimizing Interference to Primary Users in Cognitive Radio Networks: A Reinforcement Learning Approach. Proceedings of the IEEE Symposium on Computational Intelligence for Communication Systems and Networks (CIComms).

[21] Wellens M., Riihijarvi J., Mahonen P. Evaluation of Adaptive MAC-Layer Sensing in Realistic Spectrum Occupancy Scenarios. Proceedings of the IEEE Symposium on New Frontiers in Dynamic Spectrum.

[22] Xu Y., Wang W. (2013). Wireless Mesh Network in Smart Grid: Modeling and Analysis for Time Critical Communications. IEEE Trans. Wirel. Commun..

[23] Cao Y., Duan D., Cheng X., Yang L. (2014). Qos-Oriented Wireless Routing for Smart Meter Data Collection: Stochastic Learning on Graph. IEEE Trans. Wirel. Commun..

[24] El-Sherif A.A., Mohamed A. (2014). Joint Routing and Resource Allocation for Delay Minimization in Cognitive Radio based Mesh Networks. IEEE Trans. Wirel. Commun..

[25] Li J.H., Tian N.S. (2016). Analysis of the Discrete Time Geo/Geo/1 Queue with Single Working Vacation. Qual. Technol. Quantit. Manag..

[26] Hilhorst H.J., Appert-Rolland C. (2017). Mixed-Strategy Nash Equilibrium for A Discontinuous Symmetric N-Player Game. J. Phys. Math. Gen..

[27] Mnih V., Kavukcuoglu K., Silver D., Graves A., Antonoglou I., Wierstra D., Riedmiller M. (2013). Playing Atari with Deep Reinforcement Learning. arXiv.

[28] Littman M.L. (1999). A Unified Analysis of Value-Function-Based Reinforcement Learning Algorithms.

[29] Mnih V., Kavukcuoglu K., Silver D., Rusu A.A., Veness J., Bellemare M.G., Graves A., Riedmiller M., Fidjeland A.K., Ostrovski G. (2015). Human-Level Control Through Deep Reinforcement Learning. Nature.

